# Changes in body weight and composition, metabolic parameters, and quality of life in patients with type 2 diabetes treated with subcutaneous semaglutide in real-world clinical practice

**DOI:** 10.3389/fendo.2024.1394506

**Published:** 2024-07-02

**Authors:** Paola Pantanetti, Giovanni Cangelosi, Sara Alberti, Sandra Di Marco, Grazia Michetti, Gianluca Cerasoli, Marco Di Giacinti, Silvia Coacci, Nadia Francucci, Fabio Petrelli, Giuseppe Ambrosio, Roberto Grinta

**Affiliations:** ^1^ AST Fermo, Unit of Diabetology, Fermo, Italy; ^2^ AUSL Romagna, “Infermi” Hospital, Rimini, Italy; ^3^ School of Medicinal and Health Products Sciences, University of Camerino, Camerino, Italy; ^4^ Cardiology University of Perugia, Perugia, Italy; ^5^ AST Fermo, Fermo, Italy

**Keywords:** obesity, semaglutide, type 2 diabetes, body composition, real-world evidence

## Abstract

Subcutaneous once-weekly (ow) semaglutide is a recent treatment option for type 2 diabetes (T2D) and obesity, but real-world data on weight loss and associated changes in body composition, nutrients intake, and quality of life are still scarce. This observational, prospective clinical study involved all T2D patients starting ow semaglutide according to routine care between December 2021 and February 2022. Clinical information was collected after 6 months (T6) and 12 months (T12) from semaglutide initiation (T0). Bioelectrical Impedance Analysis (BIA) was performed to measure changes in body composition. Diabetes Treatment Satisfaction Questionnaire (DTSQ) and the 36 – items Short Form Health Survey (SF-36) were administered as patient-reported outcomes (PROs). Changes in continuous endpoints (weight, body composition, nutrients intake, other clinical parameters, and PROs) were assessed using mixed models for repeated measurements. Overall, 90 patients (age 63.0 ± 10.0 years; diabetes duration 7.6 ± 5.9 years; 58.9% men; HbA1c 7.7 ± 1.1%; weight 95.4 ± 19.4 Kg, BMI 34.6 ± 6.4 Kg/m^2^; 36.7% naïve to diabetes treatment, 43.3% on metformin, 10.0% on dual oral therapy, and 10.0% treated with schemes including insulin) were included in the study. After 6 months from semaglutide initiation, body weight significantly decrease by -4.69 Kg (95%CI -6.19;-3.19) (primary endpoint). After 12 months, body weight was further reduced (-5.38 Kg; 95%CI -7.79;-2.97). At BIA, fat mass was significantly reduced by 2.1 Kg after 6 months but only slightly reduced after 12 months vs. baseline; lean mass was also significantly reduced by over 3 Kg both at 6 and 12 months. Intake of all nutrients declined in the first 6 months of therapy, although only lipids reduction reached the statistical significance (-6.73 g; p=0.02). Statistically significant improvements in BMI, waist circumference, glycemic control, blood pressure and lipid profile were documented. Satisfaction with treatment (DTSQ questionnaire) and mental health (MCS score of SF-36 questionnaire) significantly increased during the follow-up. The study documented real-world benefits of semaglutide for treating obesity in T2D subjects, with important changes on clinical and patient-reported outcomes. Loss of lean mass associated with weight loss warrants attention; parallel strategies to preserve skeletal muscle and improve physical function, i.e. nutritional education and structured exercise, are of great importance.

## Introduction

Among the recent therapeutic options for the treatment of type 2 diabetes mellitus (T2D), glucagon-like peptide receptor agonists (GLP-1 RAs) play a key role. They act by increasing the concentration of the GLP-1 hormone in a physiological-pharmacological way, thus minimizing the risk of hypoglycemia (1). Furthermore, the current national and international guidelines for T2D care recommend the use of GLP-1 RA as a first-line or second-line (after metformin) therapy, taking in consideration the additional benefits on body weight and cardiorenal risk (2, 3). Since the preclinical phase, different studies documented that administration of exogenous GLP-1 at pharmacological dosages induced weight loss in addition to decrease in blood glucose levels (4). Several studies were conducted on GLP1-RAs in obesity involving patients with and without T2D and confirming that they represent a real new opportunity for chronic weight management (5, 6).

In terms of mechanisms, GLP-1 is involved in the satiety regulation at the level of the central nervous system (CNS). In fact, activation of the GLP-1 receptors reduces the brain response (in the insula, amygdala, putamen and orbitofrontal cortex areas) to food signals in subjects with obesity, with and without T2D, correlating with more or less significant reductions in food intake (7). Evidence suggests that endogenous GLP-1 is involved in the central regulation of nutrition, influencing the central reactivity to the consumption of sweet foods (8). Also, it was demonstrated that liraglutide is able to suppress the mechanisms that are activated in the CNS following visual stimulation with food (9) and that endogenous GLP-1 is responsible for the inducing effect of post-prandial satiety in the CNS in T2D (10). Furthermore, Tsuda et al. showed how the different nutrients are able to stimulate the secretion of endogenous GLP-1; therefore, how to increase the efficacy of endogenous or exogenous GLP-1 following specific diets and how exogenous GLP-1 can influence the food preferences became new key topics for further investigations (9). de Boer et al. investigated whether treatment with exogenous GLP-1 was able to improve the glycemic control of insulin-treated obese patients and whether the eating behaviors influenced the efficacy. Patients in the study showed a significant improvement in glycemic control and a significant reduction in body weight and in the dose of insulin used. The greatest reduction in body weight occurred in patients with a controlled diet, while the least reduction in weight occurred in patients with a “sensory” predominance, i.e. patients who were influenced by organoleptic aspects (10). It remained to be evaluated whether and how a treatment with pharmacological doses of GLP-1 could modify patients’ food preferences over time. In a pre-clinical study on overnourishes rats, treatment with a GLP-1 RA completely eliminated the excess weight and fat depots (11). In another study involving severely obese minipigs treated with liraglutide, body weight decreased during 7 weeks, and increased during the following 7 weeks post-treatment, documenting that the effect on appetite suppression was reversed within 4 days from treatment discontinuation (12).

Recently, a GLP-1 analogue (94% similarity with the native hormone) allowing the weekly administration, i.e. subcutaneous (sc) semaglutide, was introduced to the market for the treatment of T2D and obesity following the large research development SUSTAIN and STEP programs (13–15). SUSTAIN program documented a marked efficacy of the drug on glycemia, weight, and cardiovascular risk with maximum dose of 1 mg once weekly (14); in the STEP program semaglutide at the maximum dose of 2.4 mg once weekly achieved significant and sustained weight loss, together with improvements in cardiometabolic risk factors compared with placebo, and was generally well tolerated, with a safety profile consistent with other GLP-1RAs (15). Several real-world studies confirmed effectiveness of semaglutide on HbA1c and obesity parameters (16–22).

Our study aimed to assess clinical outcomes obtained by all T2D patients cared for by our center under routine clinical practice and treated with ow semaglutide in association with the standard nutrition and educational approach applied in our center. Specifically, we assessed real-world weight loss and the associated changes in body composition nutrients intake, and quality of life in addition to the traditional outcomes explored in other studies (metabolic control and cardiovascular risk factors).

## Materials and methods

This was a single-center, observational, prospective clinical study conducted in the Diabetes and Nutrition Clinic of Ast Fermo Asur Marche (Fermo, Italy). All patients initiating semaglutide in the trimester between December 2021 and February 2022 were included.

Inclusion criteria were: T2D diagnosis, men or women, age ≥18 years, need of therapy intensification based on the physician judgment, and signature of the informed consent.

Exclusion criteria were: intolerance or contraindications to ow semaglutide, previous GLP-1 RA or SGLT2i therapy, concomitant or suspected malignant diseases, pregnancy/breastfeeding, recent (within 3 months of enrolment visit) acute illnesses (except viral illnesses), renal impairment (eGFR<60ml/min), severe liver failure, congestive heart failure (NYHA IV classes), proliferative diabetic retinopathy, presence of cholelithiasis, chronic pancreatitis or ongoing acute pancreatitis, ketogenic diet.

According to standard care, semaglutide was prescribed at the starting dose of 0.25 mg/week during the first month of therapy, 0.50 mg/week during the second month, and 1.0 mg/week for the following months up to 12 months.

According to the clinical practice of the diabetes clinic, patients received a nutritional intervention based on the following nutrients intake: carbohydrates 45–60%, proteins 10–20%, fats 25–30%, recommended calories intake 25–30 Kcal/Kg (ideal weight).

At baseline (T0), the following patient information was collected: age, gender, T2D duration, body mass index (BMI), waist circumference (WC), glycated hemoglobin (HbA1c), fasting plasma glucose (FPG), systolic and diastolic blood pressure (SBP, DBP), total cholesterol (TOT-CHOL), high-density lipoprotein cholesterol (HDL-CHOL), low-density lipoprotein cholesterol (LDL-CHOL), triglycerides (mg/dl). Furthermore, a routine Bioelectrical Impedance Analysis (BIA) was performed (DC-430MA™, TANITA Europe BV, Amsterdam, the Netherlands) to measure body composition by exploiting the bioelectrical impedance of the body. Furthermore, evaluation of calories and nutrients intake was performed (using Metadieta Software™, Meteda srl, San Benedetto del Tronto (AP), Italy). The software allows to record dietary habits and, based on the set parameters and a standardized food atlas, automatically calculate basal metabolism, calories and grams of macronutrients.

Finally, the Diabetes Treatment Satisfaction Questionnaire (DTSQ) and the 36 – items Short Form Health Survey (SF-36) were administered as patient-reported outcomes (PROs).

Data on clinical parameters, BIA, and questionnaires were also collected after 6 months (T6) and 12 months (T12) from the ow semaglutide initiation (T0).

DTSQ has been specifically designed to measure satisfaction with diabetes treatment regimens (23). It is composed of eight items, six of which are summed in a single score ranging from 0 (very dissatisfied) to 36 (very satisfied). The remaining two items are treated individually and explore the perceived frequency of hyperglycemic and hypoglycemic episodes, with higher scores indicating a higher frequency. The Italian version of the instrument has been previously translated and validated (24).

The SF-36 Health Survey (SF-36) is one of the most widely used measures of health-related quality of life (HRQOL) and consists of 36 items covering eight dimensions: physical functioning, role limitations caused by physical health problems, bodily pain, general health perception, vitality, social functioning, role limitations caused by emotional health problems and mental health (25). These eight domains may be further aggregated into two summary measures: the physical component summary (PCS) measure and the mental component summary (MCS) measure. These aggregated scores are transformed to norm-based scores (mean, 50; SD, 10), with higher scores indicating more favorable physical functioning/psychological well-being. The SF-36 has been used in large-population studies and in many different clinical conditions, showing excellent psychometric properties (26). It has been translated and validated in several languages, including Italian (27).

The primary endpoint was the change in body weight after 6 months. Secondary endpoints were the change after 12 months in body weight, and the changes after 6 and 12 months in body composition assessed through BIA, diet attitudes, clinical parameters (BMI, WC, HbA1c, FPG, blood pressure, lipid profile), and quality of life (DTSQ and SF-36) scores.

Notifications of possible adverse events followed routine pharmacovigilance procedures and were not collected in the study.

All procedures followed were in accordance with the ethical standards of the responsible committee on human experimentation (institutional and national) and with the Helsinki Declaration. The study protocol was approved by the Ethics Committee. Informed consent was signed by all participants.

### Statistical analysis

In relation to the primary endpoint, the clinical development program of ow semaglutide showed a reduction of body weight of at least 5 kg (14). To detect a reduction of weight of at least 5 kg after 6 months with an alpha error of 0.05 and a power (beta) of 0.8 using the ANOVA model for repeated measures, a sample of at least 67 patients was needed. Based on the experience deriving from previous observational studies (16), a drop-out rate of 20% was considered; therefore at least 84 patients had to be recruited.

Descriptive data were summarized as mean and standard deviation for continuous variables and frequency and proportion for categorical variables.

Changes in continuous study endpoints (weight, body composition, nutrients intake, other clinical parameters, and PROs) were assessed by applying mixed models for repeated measurements. This method was adopted to handle missing data by means of maximum likelihood estimation, thus allowing the estimates at each follow up visit to be based on all initial cases. Results are expressed as estimated mean or estimated mean difference from T0 with their 95% confidence interval (95% CI). Paired t-test derived from linear mixed models for repeated measurements was applied for within group comparisons.

Statistical significance was declared if p-value <0.05.

The SAS software (release 9.4, SAS Institute Inc., Cary, NC, USA) was used for the analyses.

## Results

In the period between December 2021 and February 2022, 90 patients with poorly controlled T2D cared for by the center who started ow semaglutide according to routine care were identified. They had a mean age of 63.0 ± 10.0 years and a mean diabetes duration of 7.6 ± 5.9 years; 58.9% were men. Mean HbA1c was 7.7 ± 1.1% and BMI was 34.6 ± 6.4 Kg/m^2^ (**Table 1**).

**Table 1:** Characteristics of patients at the first prescription of ow semaglutide.

	Mean and standard deviation or proportion
N	90
Men (%)	58.9
Age (years)	63.0 ± 10.0
Diabetes duration (years)	7.6 ± 5.9
Weight (Kg)	95.4 ± 19.4
BMI (Kg/m^2^)	34.6 ± 6.4
HbA1c (%)	7.7 ± 1.1
FPG (mg/dl)	151.9 ± 38.2
SBP (mmHg)	136.5 ± 20.3
DBP (mmHg)	80.8 ± 14.7
Total cholesterol (mg/dl)	176.5 ± 46.8
HDL-C (mg/dL)	45.8 ± 10.8
LDL-C (mg/dL)	106.7 ± 38.4
Triglycerides (mg/dl)	173.8 ± 84.8
Last prescribed diabetes treatment before starting ow semaglutide:	
Diet only	36.7
Metformin only	43.3
Dual oral therapy	10.0
Insulin with or without other glucose-lowering agents	10.0

Before starting ow semaglutide, 33 (36.7%) subjects were treated with diet and lifestyle interventions, 39 (43.3%) were treated with metformin only, 9 (10.0%) with dual oral therapies, and 9 (10.0%) with basal insulin alone or in combination with oral antihyperglycemic agents (**Table 1**).

**Figure 1:**
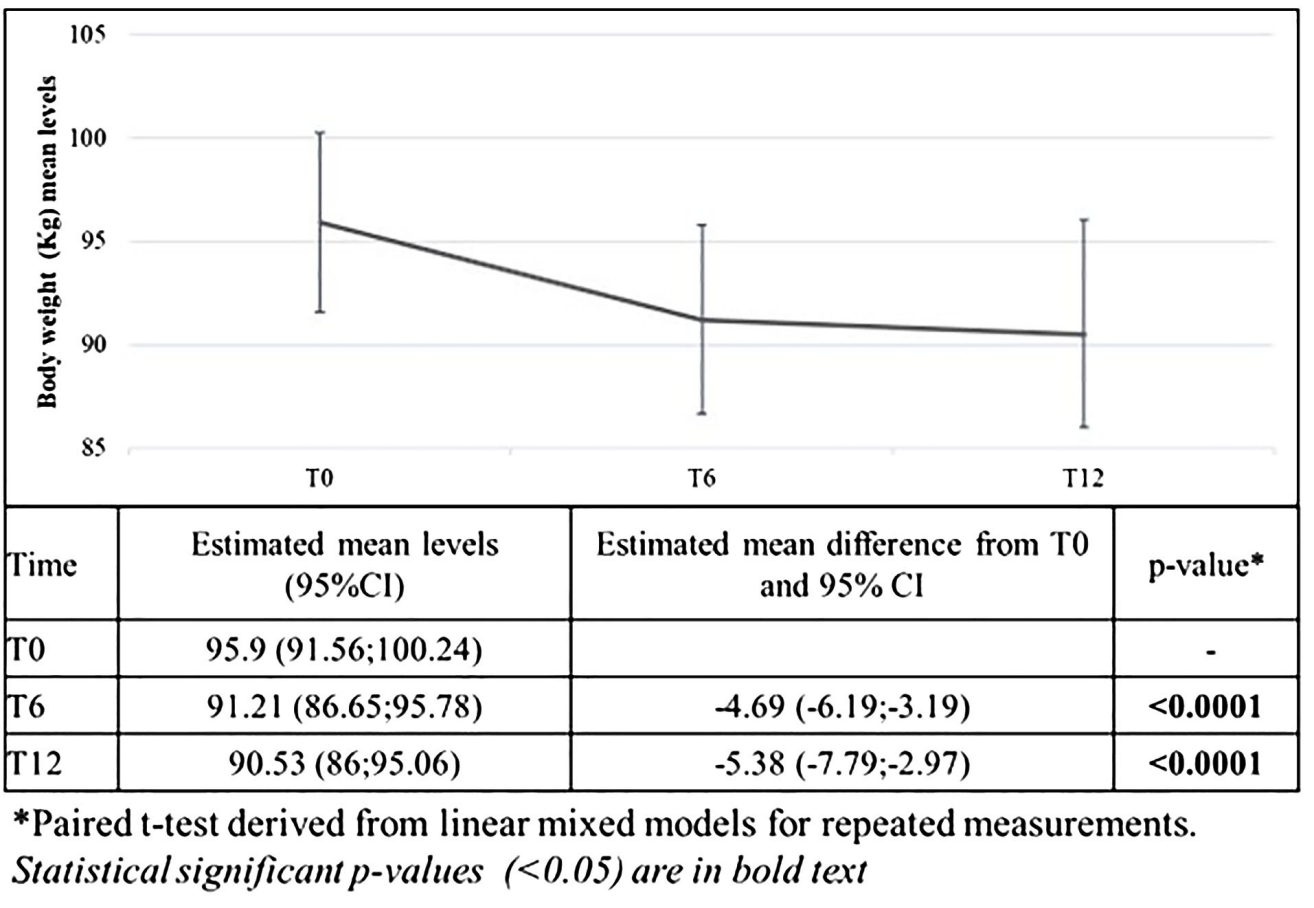
Changes in mean body weight after 6 and 12 months from the first prescription of ow semaglutide (primary endpoint).

Results relative to body weight are reported in **Figure 1**. After 6 months from the ow semaglutide initiation, body weight significantly decreased by -4.69 Kg (95%CI -6.19;-3.19) (primary endpoint). After 12 months, body weight was further reduced (-5.38 Kg; 95%CI -7.79;-2.97).

On average, body weight was reduced by 5.2 ± 4.9% after 6 months and of 5.3 ± 7.5% after 12 months. Furthermore, 45.4% of patients reached at least 5% weight reduction, and 25.0% reached at least 10% weight reduction.

Results relative to the changes in the body composition assessed through BIA are reported in **Table 2**. Overall, fat mass was significantly reduced by 2.1 Kg after 6 months but slightly reduced after 12 months; lean mass was significantly reduced by over 3 Kg both at 6 and 12 months; total body water decreased by over 2 Kg both at 6 and 12 months. Basal metabolism was reduced by 45 Kcal after 12 months.

**Table 2:** Changes in body composition assessed through BIA.

	Time	Estimated mean levels (95%CI)	Estimated mean difference from T0 and 95% CI	p-value*
**Fat mass (Kg)**	T0	35.25 (32.53;37.96)		
	T6	33.04 (30.4;35.67)	-2.21 (-3.37;-1.05)	**0.0003**
	T12	34.17 (30.99;37.34)	-1.08 (-3.82;1.66)	0.43
**Fat mass (%)**	T0	35.93 (33.96;37.89)		
	T6	35.72 (33.89;37.55)	-0.21 (-1.35;0.94)	0.72
	T12	35.8 (33.86;37.74)	-0.13 (-1.54;1.29)	0.86
**Lean mass (Kg)**	T0	59.27 (56.49;62.05)		
	T6	55.9 (53.09;58.72)	-3.37 (-4.37;-2.36)	**<0.0001**
	T12	55.74 (53;58.48)	-3.53 (-5.52;-1.54)	**0.0007**
**Lean mass (%)**	T0	64.07 (62.11;66.04)		
	T6	64.32 (62.49;66.15)	0.25 (-0.9;1.39)	0.67
	T12	64.23 (62.29;66.17)	0.16 (-1.25;1.57)	0.82
**Total Body Water (Kg)**	T0	43.22 (40.95;45.49)		
	T6	40.9 (38.79;43.02)	-2.32 (-3.29;-1.34)	**<0.0001**
	T12	40.54 (38.46;42.62)	-2.68 (-3.97;-1.39)	**<0.0001**
**Total Body Water (%)**	T0	45.04 (43.28;46.8)		
	T6	44.99 (43.48;46.5)	-0.04 (-0.9;0.81)	0.92
	T12	44.96 (43.46;46.47)	-0.07 (-0.91;0.77)	0.86
**Basal Metabolism (Kcal)**	T0	1780.48 (1707.24;1853.72)		
	T6	1753.7 (1667.28;1840.13)	-26.78 (-60.65;7.1)	0.12
	T12	1735.03 (1656.79;1813.27)	-45.45 (-81.59;-9.3)	**0.01**

*Paired t-test derived from linear mixed models for repeated measurements. Statistically significant p-values (<0.05) are in bold text.

In terms of diet attitudes, after 6 months from the first prescription of ow semaglutide, mean calories intake declined by 117 Kcal (p=0.04), but this reduction was not maintained after 12 months (**Table 3**). Intake of all nutrients declined in the first 6 months of therapy, although only lipids reduction reached the statistical significance (-6.73 g; p=0.02). At 12 months no significant changes were documented.

**Table 3:** Changes in diet attitudes assessed through dietary anamnesis (Metadieta software™).

	Time	Estimated mean levels (95%CI)	Estimated mean difference from T0 and 95% CI	p-value*
**Kcal Introduced (Kcal)**	T0	1665.09 (1560.3;1769.87)		
	T6	1547.78 (1436.21;1659.36)	-117.3 (-226.97;-7.64)	**0.04**
	T12	1648.53 (1529.73;1767.32)	-16.56 (-153.3;120.18)	0.81
**Carbohydrates (g)**	T0	217 (200;234.01)		
	T6	207.88 (190.91;224.84)	-9.13 (-30.09;11.84)	0.39
	T12	223.81 (202.39;245.23)	6.81 (-18.32;31.94)	0.59
**Proteins (g)**	T0	77.87 (72.74;82.99)		
	T6	74.72 (68.03;81.4)	-3.15 (-9.44;3.15)	0.32
	T12	77.43 (69.28;85.58)	-0.44 (-9.03;8.16)	0.92
**Lipids (g)**	T0	55.02 (51.21;58.83)		
	T6	48.30 (43.59;53)	-6.73 (-12.13;-1.32)	**0.02**
	T12	53.52 (47.7;59.35)	-1.50 (-8.17;5.17)	0.66

*Paired t-test derived from linear mixed models for repeated measurements. Statistically significant p-values (<0.05) are in bold text.

Results relative to the changes in the clinical parameters are reported in **Table 4**. Statistically significant improvements in BMI, waist circumference, HbA1c, and FBG were documented after 6 months, and sustained after 12 months. Systolic blood pressure and total cholesterol were also significantly reduced after 12 months.

**Table 4:** Changes in the obesity indices and metabolic control parameters.

	Time	Estimated mean levels (95%CI)	Estimated mean difference from T0 and 95% CI	p-value*
**BMI (Kg/m2)**	T0	34.65 (33.26;36.05)		
	T6	31.47 (28.83;34.11)	-3.18 (-5.54;-0.83)	**0.009**
	T12	32.91 (31.32;34.5)	-1.74 (-2.72;-0.77)	**0.0006**
**Waist circumference (cm)**	T0	115.6 (112.51;118.68)		
	T6	112.98 (109.73;116.23)	-2.62 (-4.29;-0.95)	**0.002**
	T12	113.22 (109.65;116.78)	-2.38 (-4.82;0.07)	0.06
**HbA1c (%)**	T0	7.71 (7.46;7.97)		
	T6	6.62 (6.37;6.87)	-1.10 (-1.43;-0.76)	**<0.0001**
	T12	6.64 (6.37;6.9)	-1.07 (-1.39;-0.76)	**<0.0001**
**FBG (mg/dl)**	T0	150.75 (142.28;159.22)		
	T6	117.51 (111.86;123.16)	-33.24 (-41.87;-24.61)	**<0.0001**
	T12	126.08 (113.66;138.51)	-24.67 (-37.29;-12.04)	**0.0002**
**SBP (mmHg)**	T0	136.25 (131.55;140.95)		
	T6	133.7 (127.4;140)	-2.55 (-9.54;4.45)	0.47
	T12	126.99 (120.72;133.26)	-9.26 (-17.11;-1.4)	**0.02**
**DBP (mmHg)**	T0	80.78 (77.31;84.25)		
	T6	76.97 (73.07;80.87)	-3.81 (-8.79;1.16)	0.13
	T12	78.16 (74.63;81.68)	-2.62 (-7.34;2.1)	0.27
**Total cholesterol (mg/dl)**	T0	174.14 (163.23;185.05)		
	T6	165.81 (151.1;180.52)	-8.33 (-23.08;6.41)	0.26
	T12	156.36 (143.88;168.85)	-17.78 (-29.67;-5.89)	**0.004**
**HDL cholesterol (mg/dl)**	T0	45.51 (42.91;48.11)		
	T6	43.91 (41.16;46.65)	-1.6 (-4.06;0.85)	0.20
	T12	43.91 (40.17;47.65)	-1.6 (-5.62;2.41)	0.43
**LDL cholesterol (mg/dl)**	T0	106.02 (96.81;115.24)		
	T6	100.65 (89.77;111.54)	-5.37 (-17.83;7.08)	0.39
	T12	96.04 (85.93;106.14)	-9.99 (-21.4;1.42)	0.09
**Triglycerides (mg/dl)**	T0	174.79 (154.6;194.97)		
	T6	153.83 (131.93;175.73)	-20.96 (-44.71;2.8)	0.08
	T12	166.27 (144.36;188.18)	-8.52 (-27.54;10.51)	0.38
**SF36 - PCS**	T0	43.82 (42.09;45.56)		
	T6	45.64 (43.46;47.83)	1.82 (-0.63;4.27)	0.14
	T12	46.65 (44.49;48.82)	2.83 (-0.02;5.68)	0.05
**SF36 - MCS**	T0	33.24 (30.6;35.89)		
	T6	37.21 (34.67;39.75)	3.97 (0.76;7.17)	**0.02**
	T12	36.5 (33.75;39.25)	3.26 (0.07;6.45)	**0.045**
**DTSQ score**	T0	27.28 (25.03;29.53)		
	T6	32.96 (30.84;35.07)	5.68 (2.59;8.77)	**0.0005**
	T12	32.29 (30.73;33.85)	5.01 (2.5;7.53)	**0.0002**

*Paired t-test derived from linear mixed models for repeated measurements. Statistically significant p-values (<0.05) are in bold text.

Results relative to the changes in the DTSQ and SF-36 scores are reported in **Table 4**. The mental component of SF-36 and the DTSQ scores significantly improved after 6 and 12 months.

## Discussion

Our study documented a weight loss of about 5 Kg after 6 and 12 months from the initiation of ow semaglutide. Prescription of ow semaglutide also produced all the expected improvements in metabolic control, lipid profile, and blood pressure previously documented both in experimental and observational studies (14–22). Improvements in these parameters were associated with better HRQoL outcomes, measured in terms of satisfaction with treatment (DTSQ questionnaire) and mental health (MCS score of SF-36 questionnaire). Interestingly, while data at 6 months were previously published (20), this was the first study to assess changes in body composition and nutrients intake following 12 months of treatment with ow semaglutide in the real-world. We found that fat and lean masses, water, basal metabolism, and intake of all nutrients (especially lipids) were reduced in the first 6 months of therapy and results were substantially maintained after 12 months.

Based on results of pivotal trials and real-world evidence in T2D subjects, an average reduction by 5 Kg in body weight and over 1.0% in HbA1c were expected (14, 17). Our results are in line with these expectations. Additional benefits on lipid profile and blood pressure were also documented in previous studies (18, 19).

Changes in dietary habits and body composition were also documented in our study, in line with previous studies. In fact, a trial conducted in 2016 showed that, after 6 months of treatment, ad libitum energy intake was substantially lower with semaglutide vs. placebo (-24%) with a corresponding loss of body weight of -5 Kg (28). In another Italian real-world study, 8 months treatment with ow semaglutide was associated with low levels of hunger, good control of eating, and meal portion size, and low levels of food cravings, as measured by the COEQ instrument (19).

Reduced appetite and energy intake, and less preference for energy‐rich foods, were also investigated in previous studies and were identified as a possible mechanism to explain the weight loss observed with once-weekly and oral semaglutide (28, 29).

In addition, pre-clinical studies highlighted that semaglutide is able to act on adipocytes and reduce visceral fat through the regulation of lipid uptake, lipid storage, and lipolysis in white adipose tissue. Besides, semaglutide may activate adipocyte browning and other marker expressions which help weight loss (30, 31).

Visceral adipose tissue is associated with increased cardiometabolic risks including insulin resistance, atherogenic dyslipidaemia, hypertension, inflammation, and coronary heart disease (32), and visceral fat reduction may be one mechanism to explain the benefits seen on cardiovascular outcomes in trials with GLP1-RA among patients with T2D (33).

Subsets from STEP 1 and SUSTAIN 8 trials documented that lean mass accounted for approximately 40% of total weight loss attributable to ow semaglutide (34, 35). A recent review highlighted that use of GLP1-RA is associated with a reduction in fat mass, with proportions of lean body mass reduction ranging between 20% and 50% of total weight lost, which is consistent with diet-induced weight loss and bariatric surgery (36). In addition, another review highlighted how the risk of sarcopenia is high in elderly people with T2D and how multifactorial intervention combining physical activity and appropriate dietary choices with the most suitable glucose-lowering drugs may play a role on preserving muscle mass and function (37).

Another aspect to be considered is that obese people with diabetes have a larger prevalence of fibrotic adipose tissue than obese people without diabetes (38). This is one of the possible reasons why weight loss in type 2 diabetes is hard to obtain. Inertia in treating obesity in T2D patients further worsens fibrosis of adipose tissue and increases CV risk (39). Therefore, early interventions are strongly recommended.

Compared with existing knowledge, what this study adds is information in the real-world about body composition after 12 months of treatment with ow semaglutide.

This study also allowed the definition of the current phenotype considered as the ideal candidate for ow semaglutide treatment in clinical practice, i.e. subjects with T2Dand other components of the metabolic syndrome (hypertension, dyslipidemia, visceral fat), with a diet prescribed as a normal part of clinical care, that alone was not sufficient to reach the body weight target. Ow semaglutide was prescribed as first-line therapy in about 1/3 of involved subjects. In fact, 34.4% were treated with diet only before starting semaglutide, suggesting the persistence of clinical inertia in initiating diabetes treatment. On the other hand, a small proportion of patients (1 out of 10) was treated with basal insulin and some patients further de-intensified insulin treatment during the follow-up (data not shown); the study enforces the evidence that use of GLP-1 RA can reduce the weight gain attributable to insulin in this population.

Increasing evidence of efficacy and safety of GLP-1 RAs may encourage health care professionals to recognize that obesity is now a treatable serious chronic disease and motivate patients to re‐engage with weight loss when previous attempts have been ineffective or unsustainable.

However, loss of lean body mass and skeletal muscle associated with weight loss induced by GLP-1RA or the other approaches warrants attention, although with any level of weight loss achieved with current strategies, a certain amount of muscle mass loss is expected, and no specific concern was reported with GLP-1 RAs (36).

Furthermore, the positive impact of ow semaglutide on treatment satisfaction and psychological well-being represents an additional, important finding. It is known that improvements in psychological well-being are a mediator for better treatment adherence, persistence in therapy in the long term, and self-reported health outcomes (40). On the other hand, regulatory agencies are still investigating the association between psychiatric disorders and all available obesity drugs. A recent study showed a lower risk association of semaglutide with suicidal ideation compared to non-GLP1R agonist anti-obesity and anti-diabetes medications (41).

Our study has strengths and limitations. The main strength was that this is the first study documenting the real-world impact of ow semaglutide on body composition in the long-term. Another strength was the prospective study design. Among limitations, lack of a control group, and lack of tolerability and safety data can be mentioned.

In conclusion, our study documented benefits of treatment with once-weekly semaglutide for treating obesity in T2D subjects managed under routine clinical conditions, with important changes on clinical and patient-reported outcomes. The documented loss of lean body mass suggests the need to implement strategies aiming at skeletal muscle preservation and physical function improvement, in addition to pharmacological approach.

## Data availability statement

The raw data supporting the conclusions of this article will be made available by the authors, without undue reservation.

## Ethics statement

The study protocol was approved by the Marche Region (Ancona, Italy) Ethics Committee, Prot. n. 2021 323 – 7818. The studies were conducted in accordance with the local legislation and institutional requirements. The participants provided their written informed consent to participate in this study.

## Author contributions

PP: Conceptualization, Data curation, Methodology, Writing – review & editing. GCa: Conceptualization, Data curation, Methodology, Writing – review & editing. SA: Writing – review & editing. SD: Writing – review & editing. GM: Writing – review & editing. GCe: Writing – review & editing. MD: Writing – review & editing. SC: Writing – review & editing. NF: Writing – review & editing. FP: Writing – review & editing. GA: Writing – review & editing. RG: Writing – review & editing.

## References

[1] Nauck MA Quast DR Wefers J Meier JJ. GLP-1 receptor agonists in the treatment of type 2 diabetes - state-of-the-art. Mol Metab. (2021) 46:101102. doi: 10.1016/j.molmet.2020.101102 33068776 PMC8085572

[2] SID-AMD Standard italiani per la cura del diabete mellito 2022 . Available online at: https://www.siditalia.it/pdf/LG_379_diabete_ed2022_feb2023.pdf (Accessed September 2023).

[3] Buse JB Wexler DJ Tsapas A Rossing P Mingrone G Mathieu C. 2019 update to: Management of hyperglycaemia in type 2 diabetes, 2018. A consensus report by the American Diabetes Association (ADA) and the European Association for the Study of Diabetes (EASD). Diabetologia. (2020) 63:221–8. doi: 10.1007/s00125-019-05039-w 31853556

[4] Drucker DJ Nauck MA. The incretin system: glucagon-like peptide-1 receptor agonists and dipeptidyl peptidase-4 inhibitors in type 2 diabetes. Lancet. (2006) 368:1696–705. doi: 10.1016/S0140-6736(06)69705-5 17098089

[5] van Bloemendaal L IJzerman RG Ten Kulve JS Barkhof F Konrad RJ Drent ML. GLP-1 receptor activation modulates appetite- and reward-related brain areas in humans. Diabetes. (2014) 63:4186–96. doi: 10.2337/db14-0849 25071023

[6] Ten Kulve JS Veltman DJ van Bloemendaal L Groot PF Ruhé HG Barkhof F. Endogenous GLP1 and GLP1 analogue alter CNS responses to palatable food consumption. J Endocrinol. (2016) 229:1–12. doi: 10.1530/JOE-15-0461 26769912

[7] Ten Kulve JS Veltman DJ van Bloemendaal L Barkhof F Drent ML Diamant M. Liraglutide reduces CNS activation in response to visual food cues only after short-term treatment in patients with type 2 diabetes. Diabetes Care. (2016) 39:214–21. doi: 10.2337/dc15-0772 26283736

[8] ten Kulve JS Veltman DJ van Bloemendaal L Barkhof F Deacon CF Holst JJ. Endogenous GLP-1 mediates postprandial reductions in activation in central reward and satiety areas in patients with type 2 diabetes. Diabetologia. (2015) 58:2688–98. doi: 10.1007/s00125-015-3754-x PMC4630252 26385462

[9] Tsuda T. Possible abilities of dietary factors to prevent and treat diabetes via the stimulation of glucagon-like peptide-1 secretion. Mol Nutr Food Res. (2015) 59:1264–73. doi: 10.1002/mnfr.201400871 25707985

[10] de Boer SA Lefrandt JD Petersen JF Boersma HH Mulder DJ Hoogenberg K. The effects of GLP-1 analogues in obese, insulin-using type 2 diabetes in relation to eating behaviour. Int J Clin Pharm. (2016) 38:144–51. doi: 10.1007/s11096-015-0219-8 PMC4733138 26597956

[11] Raun K von Voss P Gotfredsen CF Golozoubova V Rolin B Knudsen LB. Liraglutide, a long-acting glucagon-like peptide-1 analog, reduces body weight and food intake in obese candy-fed rats, whereas a dipeptidyl peptidase-IV inhibitor, vildagliptin, does not. Diabetes. (2007) 56:8–15. doi: 10.2337/db06-0565 17192459

[12] Raun K von Voss P Knudsen LB. Liraglutide, a once-daily human glucagon-like peptide-1 analog, minimizes food intake in severely obese minipigs. Obes (Silver Spring). (2007) 15:1710–6. doi: 10.1038/oby.2007.204 17636089

[13] Mahapatra MK Karuppasamy M Sahoo BM. Semaglutide, a glucagon like peptide-1 receptor agonist with cardiovascular benefits for management of type 2 diabetes. Rev Endocr Metab Disord. (2022) 23:521–39. doi: 10.1007/s11154-021-09699-1 PMC8736331 34993760

[14] Aroda VR Ahmann A Cariou B Chow F Davies MJ Jódar E. Comparative efficacy, safety, and cardiovascular outcomes with once-weekly subcutaneous semaglutide in the treatment of type 2 diabetes: Insights from the SUSTAIN 1-7 trials. Diabetes Metab. (2019) 45:409–18. doi: 10.1016/j.diabet.2018.12.001 30615985

[15] Amaro A Sugimoto D Wharton S. Efficacy and safety of semaglutide for weight management: evidence from the STEP program. Postgrad Med. (2022) 134:5–17. doi: 10.1080/00325481.2022.2147326 36691309

[16] Rudofsky G Catarig AM Favre L Grau K Häfliger S Thomann R. Real-world use of once-weekly semaglutide in patients with type 2 diabetes: Results from the SURE Switzerland multicentre, prospective, observational study. Diabetes Res Clin Pract. (2021) 178:108931. doi: 10.1016/j.diabres.2021.108931 34217773

[17] Crabtree TSJ Adamson K Reid H Barnes D Sivappriyan S Bickerton A. Injectable semaglutide and reductions in HbA1c and weight in the real world in people switched from alternative glucagon-like peptide-1 receptor agonists. Diabetes Obes Metab. (2022) 24:1398–401. doi: 10.1111/dom.14701 PMC9322019 35322528

[18] Kosiborod MN Bhatta M Davies M Deanfield JE Garvey WT Khalid U. Semaglutide improves cardiometabolic risk factors in adults with overweight or obesity: STEP 1 and 4 exploratory analyses. Diabetes Obes Metab. (2023) 25:468–78. doi: 10.1111/dom.14890 PMC10092593 36200477

[19] Di Folco U Vallecorsa N Nardone MR Pantano AL Tubili C. Effects of semaglutide on cardiovascular risk factors and eating behaviors in type 2 diabetes. Acta Diabetol. (2022) 59:1287–94. doi: 10.1007/s00592-022-01936-6 PMC9288662 35842847

[20] Volpe S Lisco G Fanelli M Racaniello D Colaianni V Triggiani D. Once-weekly subcutaneous semaglutide improves fatty liver disease in patients with type 2 diabetes: A 52-week prospective real-life study. Nutrients. (2022) 14:4673. doi: 10.3390/nu14214673 36364937 PMC9657108

[21] Menzen M Berentzen TL Catarig AM Pieperhoff S Simon J Jacob S. Real-world use of once-weekly semaglutide in type 2 diabetes: results from semaglUtide real-world evidence (SURE) Germany. Exp Clin Endocrinol Diabetes. (2023) 131:205–15. 10.1055/a-2007-2061 PMC10101737 36599459

[22] Visaria J Uzoigwe C Swift C Dang-Tan T Paprocki Y Willey VJ. Real-world effectiveness of once-weekly semaglutide from a US commercially insured and medicare advantage population. Clin Ther. (2021) 43:808–21. doi: 10.1016/j.clinthera.2021.03.003 33785221

[23] Bradley C. Diabetes treatment satisfaction questionnaire (DTSQ). In: Bradley C, editor. Handbook of psychology and diabetes. Harwood Academy Publisher, Amsterdam (1994). p. 111–32.

[24] Nicolucci A Giorgino R Cucinotta D Zoppini G Muggeo M Squatrito S. Validation of the italian version of the WHO well-being questionnaire (WHO-WBQ) and the WHO-diabetes treatment satisfaction questionnaire (WHO-DTSQ). Diabetes Nutr Metab. (2004) 17:235–43. 15575345

[25] Ware JE Jr. Sherbourne CD. The MOS 36-item short-form health survey (SF-36). I. Conceptual framework and item selection. Med Care. (1992) 30:473–83. doi: 10.1097/00005650-199206000-00002 1593914

[26] McHorney CA Ware JE Jr. Lu JF Sherbourne CD. The MOS 36-item short-form health survey (SF-36): III. Tests of data quality, scaling assumptions, and reliability across diverse patient groups. Med Care. (1994) 32:40–66. doi: 10.1097/00005650-199401000-00004 8277801

[27] Apolone G Mosconi P. The Italian SF-36 Health Survey: translation, validation and norming. J Clin Epidemiol. (1998) 51:1025–36. doi: 10.1016/S0895-4356(98)00094-8 9817120

[28] Blundell J Finlayson G Axelsen MB Flint A Gibbons C Kvist T. Effects of once-weekly semaglutide on appetite, energy intake, control of eating, food preference and body weight in subjects with obesity. Diabetes Obes Metab. (2017) 19:1242–51. doi: 10.1111/dom.12932 PMC5573908 28266779

[29] Gibbons C Blundell J Tetens Hoff S Dahl K Bauer R Baekdal T. Effects of oral semaglutide on energy intake, food preference, appetite, control of eating and body weight in subjects with type 2 diabetes. Diabetes Obes Metab. (2021) 23:581–8. doi: 10.1111/dom.14255 PMC7839771 33184979

[30] Zhu R Chen S. Proteomic analysis reveals semaglutide impacts lipogenic protein expression in epididymal adipose tissue of obese mice. Front Endocrinol (Lausanne). (2023) 21:1095432. doi: 10.3389/fendo.2023.1095432 PMC10070826 37025414

[31] Martins FF Marinho TS Cardoso LEM Barbosa-da-Silva S Souza-Mello V Aguila MB. Semaglutide (GLP-1 receptor agonist) stimulates browning on subcutaneous fat adipocytes and mitigates inflammation and endoplasmic reticulum stress in visceral fat adipocytes of obese mice. Cell Biochem Funct. (2022) 40:903–13. doi: 10.1002/cbf.3751 36169111

[32] Piché ME Tchernof A Després JP. Obesity phenotypes, diabetes, and cardiovascular diseases. Circ Res. (2020) 126:1477–500. doi: 10.1161/CIRCRESAHA.120.316101 32437302

[33] Neeland IJ Marso SP Ayers CR Lewis B Oslica R Francis W. Effects of liraglutide on visceral and ectopic fat in adults with overweight and obesity at high cardiovascular risk: a randomised, double-blind, placebo-controlled, clinical trial. Lancet Diabetes Endocrinol. (2021) 9:595–605. doi: 10.1016/S2213-8587(21)00179-0 34358471

[34] Wilding JPH Batterham RL Calanna S Davies M Van Gaal LF Lingvay I. Once-weekly semaglutide in adults with overweight or obesity. N Engl J Med. (2021) 384:989–1002. doi: 10.1056/NEJMoa2032183 33567185

[35] McCrimmon RJ Catarig AM Frias JP Lausvig NL le Roux CW Thielke D. Effects of once-weekly semaglutide vs once-daily canagliflozin on body composition in type 2 diabetes: a substudy of the SUSTAIN 8 randomised controlled clinical trial. Diabetologia. (2020) 63:473–85. doi: 10.1007/s00125-019-05065-8 PMC6997246 31897524

[36] Sargeant JA Henson J King JA Yates T Khunti K Davies MJ. A review of the effects of glucagon-like peptide-1 receptor agonists and sodium-glucose cotransporter 2 inhibitors on lean body mass in humans. Endocrinol Metab (Seoul). (2019) 34:247–62. doi: 10.3803/EnM.2019.34.3.247 PMC6769337 31565876

[37] Massimino E Izzo A Riccardi G Della Pepa G. The impact of glucose-lowering drugs on sarcopenia in type 2 diabetes: current evidence and underlying mechanisms. Cells. (2021) 10:1958. doi: 10.3390/cells10081958 34440727 PMC8393336

[38] Hajer GR van Haeften TW Visseren FL. Adipose tissue dysfunction in obesity, diabetes, and vascular diseases. Eur Heart J. (2008) 29:2959–71. doi: 10.1093/eurheartj/ehn387 18775919

[39] DeBari MK Abbott RD. Adipose tissue fibrosis: mechanisms, models, and importance. Int J Mol Sci. (2020) 21:6030. doi: 10.3390/ijms21176030 32825788 PMC7503256

[40] Nicolucci A Kovacs Burns K Holt RI Comaschi M Hermanns N Ishii H. Diabetes Attitudes, Wishes and Needs second study (DAWN2™): cross-national benchmarking of diabetes-related psychosocial outcomes for people with diabetes. Diabetes Med. (2013) 30:767–77. doi: 10.1111/dme.12245 23711019

[41] Wang W Volkow ND Berger NA Davis PB Kaelber DC Xu R. Association of semaglutide with risk of suicidal ideation in a real-world cohort. Nat Med. (2024) 30:168–76. doi: 10.1038/s41591-023-02672-2 PMC11034947 38182782

